# Deep learning initialized compressed sensing (Deli-CS) in volumetric spatio-temporal subspace reconstruction

**DOI:** 10.1007/s10334-024-01222-2

**Published:** 2025-02-01

**Authors:** S. Sophie Schauman, Siddharth S. Iyer, Christopher M. Sandino, Mahmut Yurt, Xiaozhi Cao, Congyu Liao, Natthanan Ruengchaijatuporn, Itthi Chatnuntawech, Elizabeth Tong, Kawin Setsompop

**Affiliations:** 1 Department of Radiology, Stanford University, Stanford, CA USA; 2 Department of Clinical Neuroscience, Karolinska Institute, Stockholm, 17177 Sweden; 3 Department of Electrical Engineering and Computer Science, Massachusetts Institute of Technology, Cambridge, MA USA; 4 Department of Electrical Engineering, Stanford University, Stanford, CA USA; 5 Center of Excellence in Computational Molecular Biology, Chulalongkorn University, Bangkok, Thailand; 6 Center for Artificial Intelligence in Medicine, Chulalongkorn University, Bangkok, Thailand; 7 National Nanotechnology Center, National Science and Technology Development Agency, Pathum Thani, Thailand

**Keywords:** Deep learning, Magnetic resonance imaging, Algorithms, Brain, Image processing (computer-assisted)

## Abstract

**Object:**

Spatio-temporal MRI methods offer rapid whole-brain multi-parametric mapping, yet they are often hindered by prolonged reconstruction times or prohibitively burdensome hardware requirements. The aim of this project is to reduce reconstruction time using deep learning.

**Materials and methods:**

This study focuses on accelerating the reconstruction of volumetric multi-axis spiral projection MRF, aiming for whole-brain T1 and T2 mapping, while ensuring a streamlined approach compatible with clinical requirements. To optimize reconstruction time, the traditional method is first revamped with a memory-efficient GPU implementation. Deep Learning Initialized Compressed Sensing (Deli-CS) is then introduced, which initiates iterative reconstruction with a DL-generated seed point, reducing the number of iterations needed for convergence.

**Results:**

The full reconstruction process for volumetric multi-axis spiral projection MRF is completed in just 20 min compared to over 2 h for the previously published implementation. Comparative analysis demonstrates Deli-CS’s efficiency in expediting iterative reconstruction while maintaining high-quality results.

**Discussion:**

By offering a rapid warm start to the iterative reconstruction algorithm, this method substantially reduces processing time while preserving reconstruction quality. Its successful implementation paves the way for advanced spatio-temporal MRI techniques, addressing the challenge of extensive reconstruction times and ensuring efficient, high-quality imaging in a streamlined manner.

**Supplementary Information:**

The online version contains supplementary material available at 10.1007/s10334-024-01222-2.

## Introduction

Recent advances in spatio-temporal MRI techniques have reduced scan durations but struggle with extended reconstruction times. These methods leverage efficient k-space encoding and innovative temporal subspace reconstruction techniques [1–14], along with precise regularization, for high-quality reconstructions that mitigate noise and undersampling artifacts, even at high acceleration rates. However, extended reconstruction times, especially for high-resolution volumetric imaging, remain a notable challenge, hindering the integration of these efficient techniques into clinical practice despite their impressive acquisition efficiency.

Consider the *Tiny Golden Angle Shuffling Spiral Projection Imaging Magnetic Resonance Fingerprinting* (TGAS-SPI-MRF [15]) volumetric acquisition, which serves as the focal point of this study. In previous research by Cao et al. [15], this method introduced an optimized trajectory enabling whole-brain multi-parametric mapping at a remarkable 1 mm^3^ resolution within just 2 min of acquisition time. The technique employs a spiral trajectory, multi-flip angle, fingerprinting sequence, yielding a time series of highly undersampled data. Reconstruction is performed through subspace-basis reconstruction, reducing the degrees of temporal freedom from 500 time points to just five subspace coefficients. Together with strong regularization in the form of locally low rank (LLR) constraints [16], and an iterative reconstruction implemented in BART [17].

This manuscript centers on the refinement of the reconstruction process for 2-min MRF data. The BART implementation used by Cao et al. necessitated approximately two hours and 10 min of computation, utilizing up to 80 CPU threads and peaking at around 140 GB of resident memory usage during the iterations. The challenge lies in the problem’s high dimensionality, which limits out-of-the-box GPU utilization for potential speed improvements. Despite BART’s default optimizations for reconstruction speed, including the use of the theoretically optimal Fast Iterative Shrinkage-Thresholding Algorithm (FISTA) [18] for solving the LLR-regularized optimization, as well as the combination of the *Toeplitz Point Spread Function *[19, 20] and the *spatio-temporal kernel *[12, 21] to reduce the per-iteration compute time, extensive pre-calculation remains necessary, demanding up to 400 GB of resident memory.

Concurrently with the development of fast spatio-temporal MRI acquisition, significant progress has been made in utilizing deep learning for image reconstruction for acquisitions with high rates of acceleration [22–27]. These methods commonly leverage an *unrolled* deep learning architecture, where the algorithm alternates between network inference and a data-consistency step akin to compressed sensing like iterative methods [28–32].

However, utilizing such unrolled physics-driven methods can be challenging depending on the dimensionality of the problem of interest. As an example, in this project, the image volume is of dimensions 256 × 256 × 256 × 5, where 256 × 256 × 256 is the matrix size of the acquisition, and 5 denotes the number of subspace coefficients (that are needed to represent the temporal characteristics of the underlying signal) [6, 10–12, 15, 25].

This, along with using multiple receive channels for the acquired data across k-t-space dramatically increases the dimensionality of the reconstruction problem. The memory requirement for the image volume alone is 256 × 256 × 256 × 5 × 64 bits (complex values stored as two 32-bit floats) = 600 MB, expansion into receive channels using the forward operator expands the memory usage by another factor of 10 = 6 GB. This is just to store the image expanded into its coil projections, which is a necessary intermediate step in the operation. Not to mention the memory required for the NUFFT itself and the projection from subspace to time-points. This problem is also not separable in the 3D spatial dimensions, as the k-space trajectory is designed to spread aliasing along all spatial dimensions, unlike acquisitions with rectilinear trajectories where the problem can be divided along the readout dimension. The size of the data makes such unrolled physics-driven reconstructions extremely computationally challenging to both train and deploy without specialized hardware.

With these constraints in mind, this work proposes *Deep Learning Initialized Compressed Sensing* (Deli-CS). The goal is rapid and highly compute-efficient subspace reconstruction of TGAS-SPI-MRF with the ultimate aim of clinical deployment. This framework targets less than 6 GB peak GPU memory usage and a reconstruction of the 2-min TGAS-SPI-MRF acquisition that is comparable in quality to iterative LLR reconstruction. Unlike other DL methods that have been developed for improving image quality, the aim of this project was strictly decreased reconstruction time and hardware constraints, as good image quality was already demonstrated for this application by Cao et al. [15].

The manuscript first outlines optimizations to the traditional LLR reconstruction implemented in a GPU-efficient manner. Next, the complete Deli-CS framework is described, where a fast initial reconstruction is fed into a neural network that attempts to predict the final reconstruction. Data consistency in Deli-CS is enforced through ‘compressed sensing certification’ which ought to converge faster with the DL initialization as the starting point should be closer to the theoretical minimum of the cost function [33, 34].

## Materials and methods

### Theory

Spatio-temporal subspace-reconstruction is generally posed as a linear inverse problem where the acquisition operator, **A ∈ **ℂ^MTC × NK^, models the transformation from the input subspace coefficient images (**x ∈ **ℂ^NK × 1^) to the data acquired, **b ∈ **ℂ^MTC × 1^. With T being the total number of TRs, K denoting the number of coefficient images, or the rank of the low-rank subspace utilized, N the number of voxels in image space, M the number of k-space samples per TR, and C the number of coils.. Then, the acquisition operator **A** is as follows:

1 $${\text{A = FS}}\Phi$$

Here, **F ∈ **ℂ^MTC × NTC^ denotes the forward NUFFT operator [35], **S ∈ **ℂ^NTC × NT^ the projection onto coil sensitivity maps according to the SENSE [36, 37] model and **ɸ ∈ **ℂ^NT × NK^ the linear subspace.

The low-rank subspace **ɸ** is estimated by taking the Singular Value Decomposition (SVD) of the signal dictionary generated from Bloch simulations using realistic brain tissue parameters.

The forward operation **ɸx** recovers the TR images of the TGAS-SPI-MRF acquisition.

A rank *K* = 5 subspace was deemed sufficient in capturing the signal variation as per Cao et al. [15].

The linear inverse problem used to solve the subspace reconstruction is as follows:

2 $$ \min_{\text{x}} \left( {\frac{1}{2}||{\text{Ax}} - {\text{b}}||_{2}^{2} + \;\lambda {\text{LLR}}\left( {\text{x}} \right)} \right)$$

Here, *λ* is the regularization value and LLR denotes the locally low-rank constraint.

The LLR operator breaks the reconstructed volume up into blocks, and for each block performs a singular value decomposition (SVD). In our implementation of the subspace reconstruction, the SVD is applied across the subspace coefficients. The singular values are thresholded based on the *λ* weighting, enforcing local low-rankedness among voxels within each block. The value of *λ* for this work was chosen based on the proposed value from Cao et al. [15]. Since the optimal *λ* is determined by the image and not by initialization of the iterative reconstruction, we were confident that the same *λ* would work equally well in our work with the deep-learning initialized reconstruction.

### Data acquisition

**Fig. 1:**
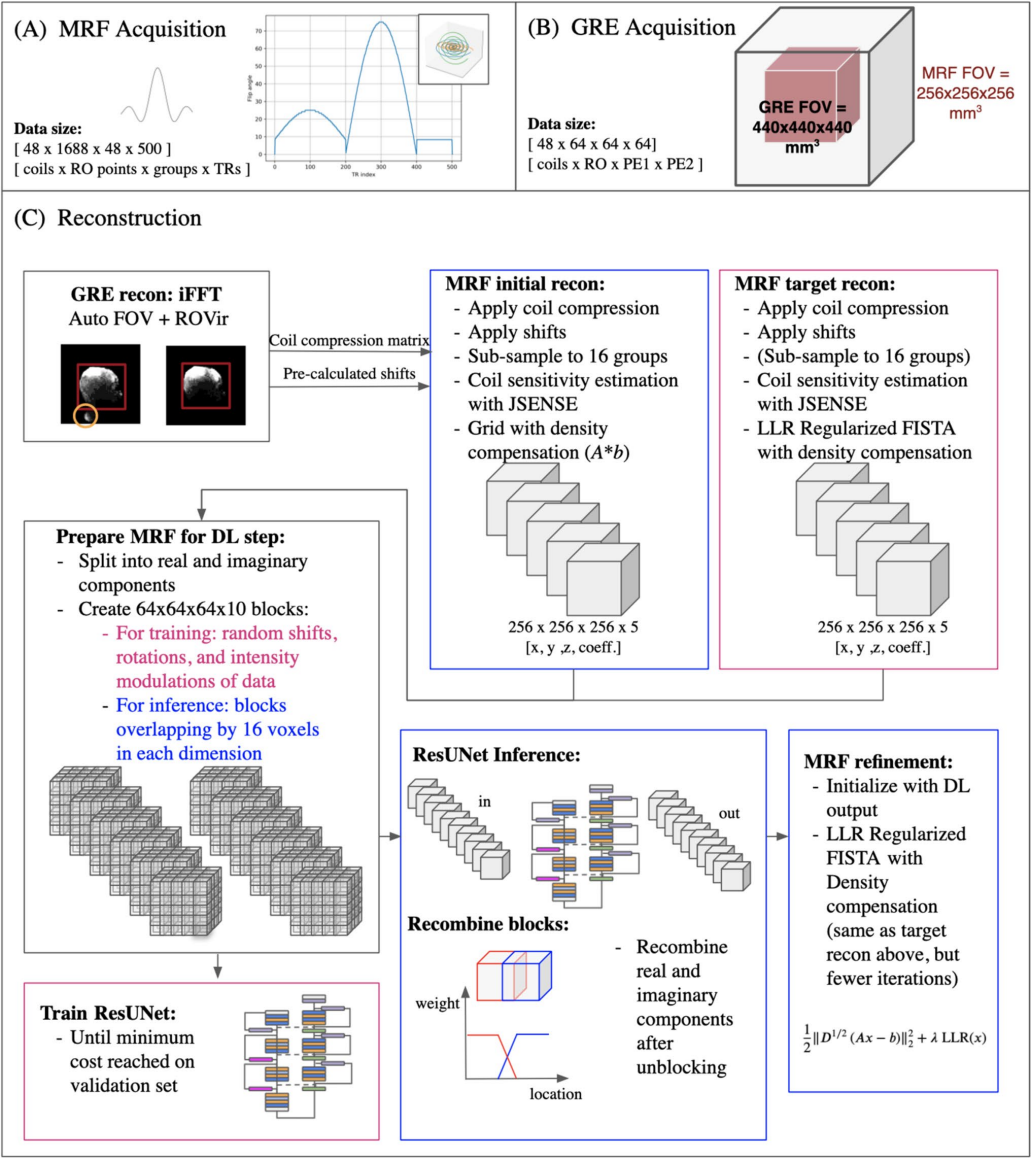
The Deli-CS pipeline includes two sequences: MRF depicted in the box **A** that shows one MRF group, which consists of an inversion pulse followed by a 500 TR acquisition train with flip angles varying from 10 to 75 degrees acquired with a spiral sampling pattern. The second sequence is a simple large FOV GRE sequence depicted in the box **B** by showing how the FOV was set up in comparison to the MRF FOV. Box **C** depicts the reconstruction pipeline. Starting with the GRE reconstruction and autoFOV to reduce signals outside the MRF FOV (depicted as a red square) and ROVir to further eliminate signals outside the FOV (orange circle). Then the MRF data is processed using the coil compression matrix and shifts estimated from the GRE. Elements in blue are only used in the inference pipeline, whereas elements in magenta were only used when training the network

The TGAS-SPI-MRF acquisition proposed by Cao et al. [15] involves an initial adiabatic inversion pulse followed by a 500 TR long readout train (TI/TE/TR = 20/0.7/12 ms) with varying flip angles (10 to 75 degrees) and a 3D center-out spiral trajectory (Fig. 1A). A water-exciting rectangular RF pulse with a duration of 2.38 ms to suppress the fat signal [38] was used. No gradient delay correction was applied. The gold standard acquisition used 48 readout trains to fully fill k-space. Only 16 readout trains (*R* = 3) were used for the accelerated 2-min acquisition.

Additionally, a 20 s, low-resolution (6.9 mm^3^) gradient echo (GRE) pre-scan with a large field-of-view (FOV) of 440 mm was performed (Fig. 1B). This pre-scan was used for automatic FOV shifting and to pre-calculate a coil compression matrix, such that any signal originating from outside the TGAS-SPI-MRF FOV after shifting (e.g. shoulders) could be removed using region-optimized virtual coil compression (ROVir) [39].

### Memory optimization

This work uses the Python-based SigPy [40] framework for its ease-of-use reconstruction whilst retaining control over GPU memory management. Reduced memory burden improves both standard physics-driven iterative reconstruction and the Deli-CS pipeline. To reduce the memory requirements of **A**, the following optimizations are made:

First, the forward model **A** is split into smaller sub-models so that each sub-model can be evaluated on the GPU on each subspace, K, and each channel, C, independently. This is possible since the data per channel do not interact with each other (and are simply stacked), and because of the second optimization, which avoids expanding into the dimension, T, which is ~ 100 times larger than the subspace dimension, K., The order of the subspace operator **ɸ** and the transform from image to k-space is reversed. This is possible because neither **S** nor **F** affects the temporal dimension that **ɸ** acts on.

These two optimizations together result in the following forward model:

3 $$A = \left[ {\begin{array}{*{20}c} {A_{{\left( {1,1} \right)}} } & {A_{{\left( {2,1} \right)}} } & \cdots & {A_{{\left( {K,1} \right)}} } \\ {A_{{\left( {1,2} \right)}} } & {A_{{\left( {2,2} \right)}} } & \cdots & {A_{{\left( {K,2} \right)}} } \\ \vdots & \vdots & {} & \vdots \\ {A_{{\left( {1,C} \right)}} } & {A_{{\left( {2,C} \right)}} } & \cdots & {A_{{\left( {K,C} \right)}} } \\ \end{array} } \right]$$

With

4 $${\text{A}}_{{\left( {k,c} \right)}} = \Phi_{k} {\text{F}}\left( {1 + 2 + ... + T} \right){\text{S}}_{c}$$

Here, **ɸ**_k_ ∈ ℂ^MT × MT^ denotes the transform from one subspace component in k-space to its counterpart time series in k-space, **F**_1,2,…,T_ ∈ ℂ^MT × N^ is a NUFFT mapping image space to k-space locations for all TR’s combined, and **S**_c_ ∈ ℂ^N × N^ denotes the c^th^ coil-sensitivity map. The individual smaller sub-model linear operators, **A**_k,c_ ∈ ℂ^MT × N^, can be evaluated one-by-one. The inputs to each sub-model linear operator, **x**_k_ ∈ ℂ^N × 1^, is first transferred to GPU memory before applying the operator, **A**_k,c_**x**_k_, and the resulting outputs (k-space time courses weighted by k) are is then summed over k (columns in the matrix **A**), and transferred back into CPU memory before the stacking over coils, c (rows in the matrix **A**) to keep the memory burden on the GPU as low as possible.

The modified linear operator only utilizes approximately 13 GB of peak CPU memory and 4.7 GB of peak GPU memory, compared to 140 GB peak CPU memory for the BART implementation. BART uses Toeplitz embedding, which reduces per-iteration computation time as the NUFFT’s in the acquisition model can be replaced by FFT’s, but increases memory usage and requires a pre-calculation, which had a peak memory usage of > 400 GB.

However, despite the improvement in memory utilisation, the modified linear operator proposed in (3) increases the per iteration speed of FISTA when solving (2) for a 2-min TGAS-SPI-MRF acquisition in SigPy on a single GPU from approximately 26 s for BART on the CPU to approximately 58 s (a 45% reduction in efficiency). These times were observed on a Linux workstation with 80 threads on Intel Xeon Gold 5320 CPUs at 2.20 GHz and an NVIDIA RTX A6000 GPU. Note that this performance is expected to vary between hardware and that the reduction in per-iteration speed is counteracted by the lower memory burden.

### Density compensation

Having achieved considerably lower memory usage, the next optimization targets improving the iterative convergence. The acquisition operator **A** is ill-conditioned, yielding slow iterative convergence. Assuming 25 s per iteration and 300 iterations, which was used by Cao et al. [15], this results in approximately 2 h 10 min required to reconstruct data. To improve the conditioning of **A**, first Pipe-Menon density compensation [41] was integrated into the optimization (Eq. 2) as described in [19, 20], yielding the following optimization:

5 $$\min_{\text{x}} \left( {\frac{1}{2}||{\text{D}}^{{{1 \mathord{\left/ {\vphantom {1 2}} \right. \kern-0pt} 2}}} \left( {{\text{Ax}} - {\text{b}}} \right)||_{2}^{2} + \;\lambda {\text{LLR}}\left( {\text{x}} \right)} \right)$$

Here, **D** is the Density Compensation array designed to target **F**_1 + 2 + … + T_ in Eq. 3 so that A*DA has better conditioning.

### Field of view processing

Caution is required when using a tight FOV in TGAS-SPI-MRF to avoid artifacts caused by signals originating outside the FOV, such as those from the shoulders and neck. This can be overcome by utilizing automatic FOV shifting and FOV size adaption, for example, the approach proposed in Baron et al. [20].

Since the MRF sequence acquires a fixed FOV around isocenter it is sensitive to subject positioning. An autoFOV algorithm was implemented to ensure that all anatomy was inside the FOV for the MRF. The autoFOV feature also allows speed up scanning because the FOV does not need to be set up manually.

The automatic FOV centering was done by reconstructing the calibration GRE image and performing a sum-of-squares combination of data from the multiple receive coils. The image was then flattened by taking a maximum-intensity projection through the sagittal plane. The resulting 2D image was then smoothed, binarized, and a bounding box was calculated around the largest continuous area. A shift was then calculated to ensure the top and front of the bounding box was within the target field-of-view.

The GRE data was pre-whitened before ROVir was used to calculate a weighting of the 48 input channels that minimizes signal outside the MRF FOV (referred to as the interference region [39]). Eight virtual ROVir coils with the most signal in the interference region were excluded. SVD compression to 10 virtual channels of the remaining 40 channels was then performed. The complete coil compression matrix containing coil whitening, ROVir, and SVD compression was calculated based on the GRE and used for compression of the TGAS-SPI-MRF data. Coil sensitivity maps used in the MRF reconstruction were estimated from the MRF data itself using JSENSE [42].

The reconstruction, automatic FOV shifting, and coil processing matrix calculation for the GRE data takes less than 30 s and can be run while the TGAS-SPI-MRF data is being acquired.

### Deli-CS

The Deli-CS pipeline consists of the following steps:

1. *Traditional Reconstruction*. As a first step, an approximate reconstruction is performed by gridding the acquired data A*b. This reconstruction, the input to the next step, suffers from severe temporal-aliasing artifacts and increased noise, in particular in the lower energy subspace components.
2. *Block-based Data-Driven Deep Learning*. A deep learning network (ResUNet [43]) was trained to denoise the input image. The model is both trained and deployed in a *block-wise* manner to reduce the memory and training data requirements and is consequently *not* integrated with data consistency terms in an unrolled manner. The block-based processing proposed in this step allows the deep learning model to be trained and deployed efficiently with 5 GB of GPU memory.
3. *Compressed Sensing Certification*. Since the above network is block-based and data-driven, the inferred reconstruction is susceptible to hallucinations. The inferred result is therefore used to initialize Eq. 5 solved with an iterative reconstruction. By initializing with the inferred result, the number of iterations required to converge is significantly reduced. Additionally, the resulting image satisfies the same convergence criterion as the traditional reconstruction achieved when solving Eq. 5.

**Fig. 2:**
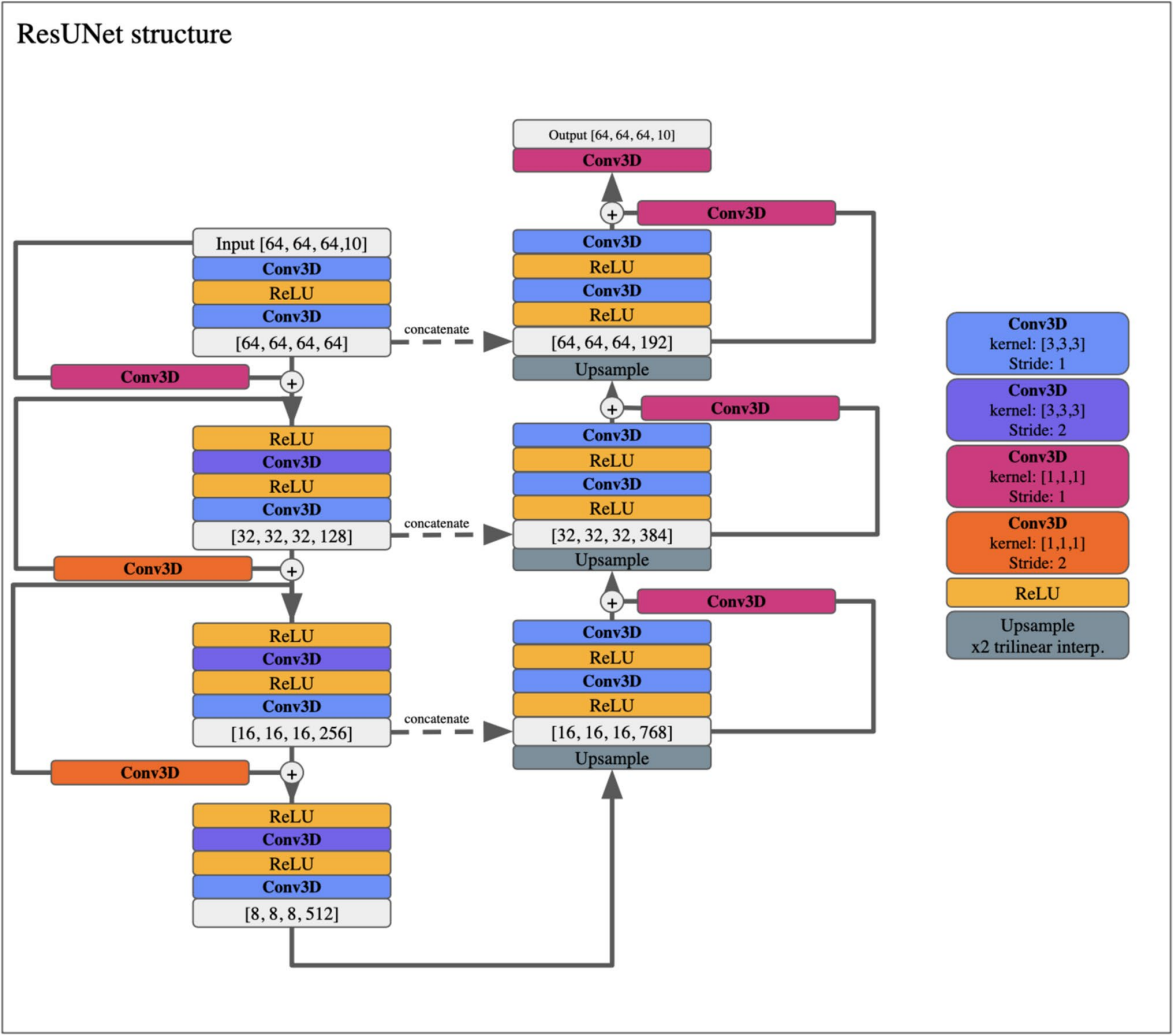
The network structure of the ResUNet structure used in Deli + CS. The light gray boxes show the data size at various layers of the network, where the first three numbers are the spatial dimensions, and the fourth number is the number of feature channels. The convolutions with kernel size 1 in the skip connections match the input and output dimensions so that they can be added together after each convolutional layer

The full Deli-CS reconstruction pipeline is depicted in Fig. 1C, and its DL network structure is shown in Fig. 2.

### Experiments

Data from 14 healthy volunteers were acquired on a 3 T Premier MRI scanner (GE Healthcare, Waukesha, WI) and a 48-channel receive coil. GRE and TGAS-SPI-MRF data were acquired. The TGAS-SPI-MRF acquisition time was 6 min, acquired resolution was 1 mm^3^, and FOV was 220 mm^3^. The data was retrospectively sub-sampled to simulate a 2-min acquisition. The data were partitioned as 10 training, 2 validation and 2 testing subjects. The technique was additionally tested on three sets of patient data (all male, ages 28, 49, and 75) acquired as additional scans to the standard of care protocols using two different 3 T Signa Premier scanners. The patients were scanned with a prospectively accelerated 2-min TGAS-SPI-MRF protocol and thus no 6-min reference comparison was possible for these cases.

All human data were acquired with informed consent using protocols approved by the local institutional review board.

Coil sensitivity maps were estimated with JSENSE [42] from the time-averaged acquisitions. The subspace basis was generated from a simulated signal dictionary, and template matching was used to estimate the T1, and T2 parametric maps [1]. The tissue parameters used to generate the signal dictionary were:

$$\begin{aligned} T_{1} \in \left\{ {20,\;40,...,\;3000} \right\}\;\;\;\;\;\;\;\;\;\;\;\;\;\;\;\;\;\;\; \cup \\ \left\{ {3200,\;3400,\;3600,...,\;5000} \right\} \\ \end{aligned}$$

$$\begin{aligned} T_{2} \in  \left\{ {10,\;20,\;14,...,\;200} \right\}& \cup \\ \left\{ {220,\;240,\;260,...,\;1000} \right\}&\cup \\ \left\{ {1050,\;1100,\;1150,...,\;2000} \right\}&\cup \\ &\left\{ {2100,\;2200,\;2400,...,\;4000} \right\} \\ \end{aligned}$$

The reported λ values for all reconstructions are after **D**^1/2^**b** in Eq. 5 was normalized to have unitary l_2_ norm.

A gold standard LLR reconstruction was performed on the 6-min data with an LLR block size of 8 and a λ value of 3 × 10^–5^ (based on similar values in previous work [15]) with 40 FISTA iterations. The matrix size was 256 × 256 × 256 × 5. This gold standard reconstruction is labelled MRF target recon in Fig. 1 and was only used for reference to compare the performance of the different reconstruction approaches of the retrospectively undersampled 2-min case.

The retrospectively undersampled 2-min data LLR reconstruction was also performed using a λ value of 5 × 10^–5^ with 40 FISTA iterations. In both the 6-min and 2-min cases this was sufficient for convergence. These data were used as target reconstructions when training the Deli-CS network.

For training the Deli-CS network, non-overlapping blocks of dimensions 64 × 64 × 64 × 5 were extracted from the initial adjoint reconstruction and the reference reconstruction of the 2 min acquisition. For data augmentation, random flips (mirroring of the block in the x, y, and z, direction), transposes (swapping the order of the x, y, and z dimensions), and spatial shifts of random integer voxel steps in each direction were performed on the whole volume, before augmentation of the absolute scaling (between 50 and 100% of the original scale) was performed on each block. The block size was chosen to fit in GPU memory, but no hyper-parameter search was performed to assess the block size’s impact on network performance.

Before augmentation, the volume was normalized to have l_2_-norm = 1. If any block’s 0th order Fourier coefficient’s standard deviation was below 0.3, that block was discarded. This filter ensures that the network avoids learning from regions with no signal variation (e.g. background only blocks), which provide no useful information, but significantly increases the training time of the network. The blocks are subsequently split into real and imaginary components, and concatenated blockwise along the subspace dimension. A total of 623 training blocks from 10 subjects and 152 validation blocks from 2 subjects were used. These blocks were piped into a ResUNet [43] with 3D convolutions, where the channel dimension corresponds to the subspace dimension. The ResUNet utilized 3 residual encoding blocks and 3 residual decoding blocks with a filter size of 3 × 3 × 3 and ReLU activation. The total number of trainable parameters was 23.8 million. The model was implemented in PyTorch Lightning [44] and trained for 540 epochs (stopping criteria defined by when minimal l_1_-loss on the validation set was reached, see Supplemental Figure S1 for convergence plot.) using the Adam optimizer [45] with a learning rate of 1 × 10^–5^. The training utilized 5 GB of GPU memory.

To avoid edge effects during inference, blocks were extracted from the initial reconstruction with a 16-voxel overlap in all dimensions. The resulting blocks were combined by applying a linear cross-blend operation, where the overlapping regions were averaged using a weighted average with weights linearly increasing from the edge of the overlapping region to the centre of the block (Fig. 1C).

For the final step of Deli-CS, the model prediction is used to initialize Eq. 5 and compared with the standard initialization of A*b. The iterative reconstruction used for refinement utilized the same parameters as the reference 2 min reconstruction.

The T1 and T2 values for different numbers of reconstruction iterations were assessed in a gray matter and white matter mask generated by FSL FAST [46] after brain segmentation using FSL BET [47].

## Results

**Fig. 3:**
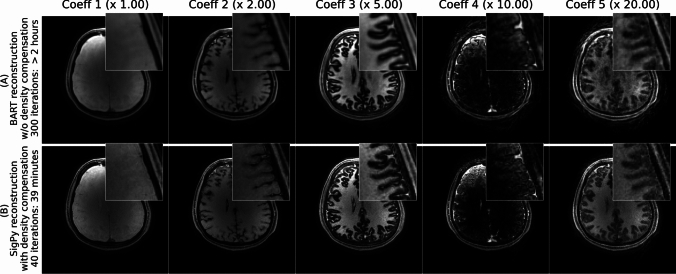
This figure compares a prior method by Cao et al.[15] **A** to the updated reconstruction **B** that includes density compensation to significantly improve the conditioning of the problem, resulting in both much faster iterative convergence and improved sharpness compared to not using density compensation. The data shown here is for a 2 min acquisition. The number above each column shows how much the intensity of each coefficient image has been multiplied for visualization

As seen in Fig. 3, using density compensation and GPU-optimized processing improves both reconstruction quality (sharpness in particular) and time compared to the prior work that used LLR without density compensation [15]. With density compensation, the iterates mostly denoise the image, whereas without density compensation the iterates deblur the image. Because of this, the BART reconstruction looks slightly blurrier as it may not have reached complete convergence in 300 iterations.

**Fig. 4:**
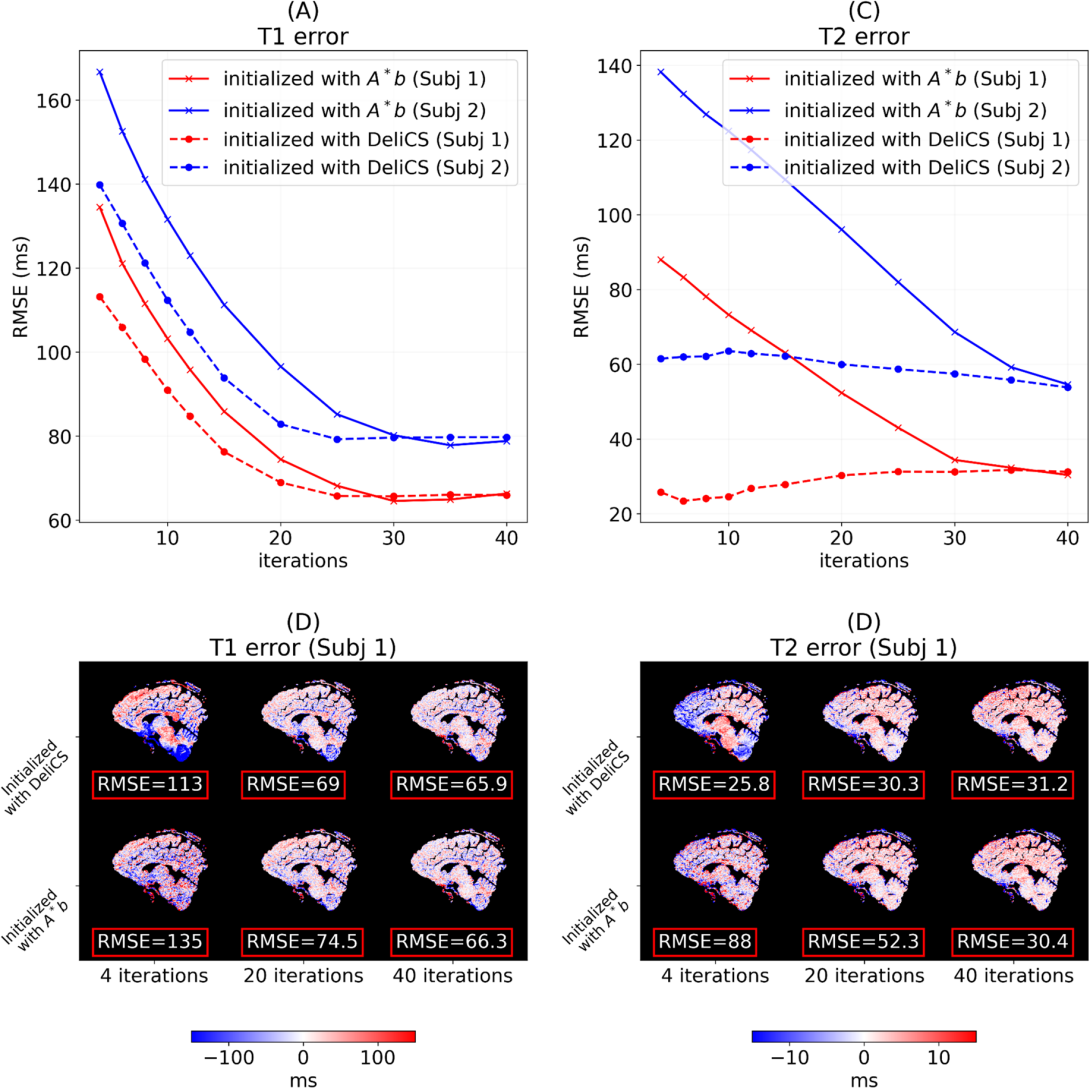
The estimated T1 RMSE values (**A**, **B**) and especially T2 RMSE values (**C**, **D**) within the white matter and gray matter in the two test subjects are shown to converge faster for the Deli-CS approach than standard initialization of the FISTA reconstruction

As shown in Fig. 4A and B, the Deli-CS initialized prediction of T1 maps had a slightly lower RMSE error than the reference 2-min LLR reconstruction after 4 iterations and after 20–30 iterations both reconstructions reached convergence. However, for the Deli-CS initialized reconstruction, the iterations mostly removed local biases introduced by the Deli-CS step (see blotch-like errors in the 4 iteration results, mostly in the lower part of the brain, in Fig. 4B and D), whereas the conventional iterative reconstruction mostly denoised the T1 maps. T2 maps estimation on the other hand benefited greatly from the Deli-CS initialization as can be seen in Fig. 4C and D. The convergence of the T2 values for the conventional reconstruction required the full 40 iterations, whereas the Deli-CS initialized estimates had low error from the beginning and the iterative refinement changed the error from bias to more noise-like error. As RMSE does not distinguish between noise-like and bias-like errors, the iterative plot (Fig. 4C) does not directly convey quality convergence in the T2 case.

After 20 iterations of refinement, the RMSE error of the T1 and T2 maps were similar to that of 40 iterations of adjoint-initialized reconstruction, providing a 2 × acceleration factor on top of what the memory efficient and density compensated SigPy implementation of the algorithm already generated. Since the refinement step is the longest step in the Deli-CS pipeline, this improvement dominates the overall reconstruction time improvement. The Deli-CS initial adjoint reconstruction took approximately 20 s; the Deli-CS model inference took approximately 20 s; and the final refinement step with 20 iterations took approximately 19 min and 20 s. Compared with the reference iterative reconstruction, which took 39 min, the Deli-CS approach enables approximately > 2 × faster processing times with 50% fewer iterations in total.

**Fig. 5:**
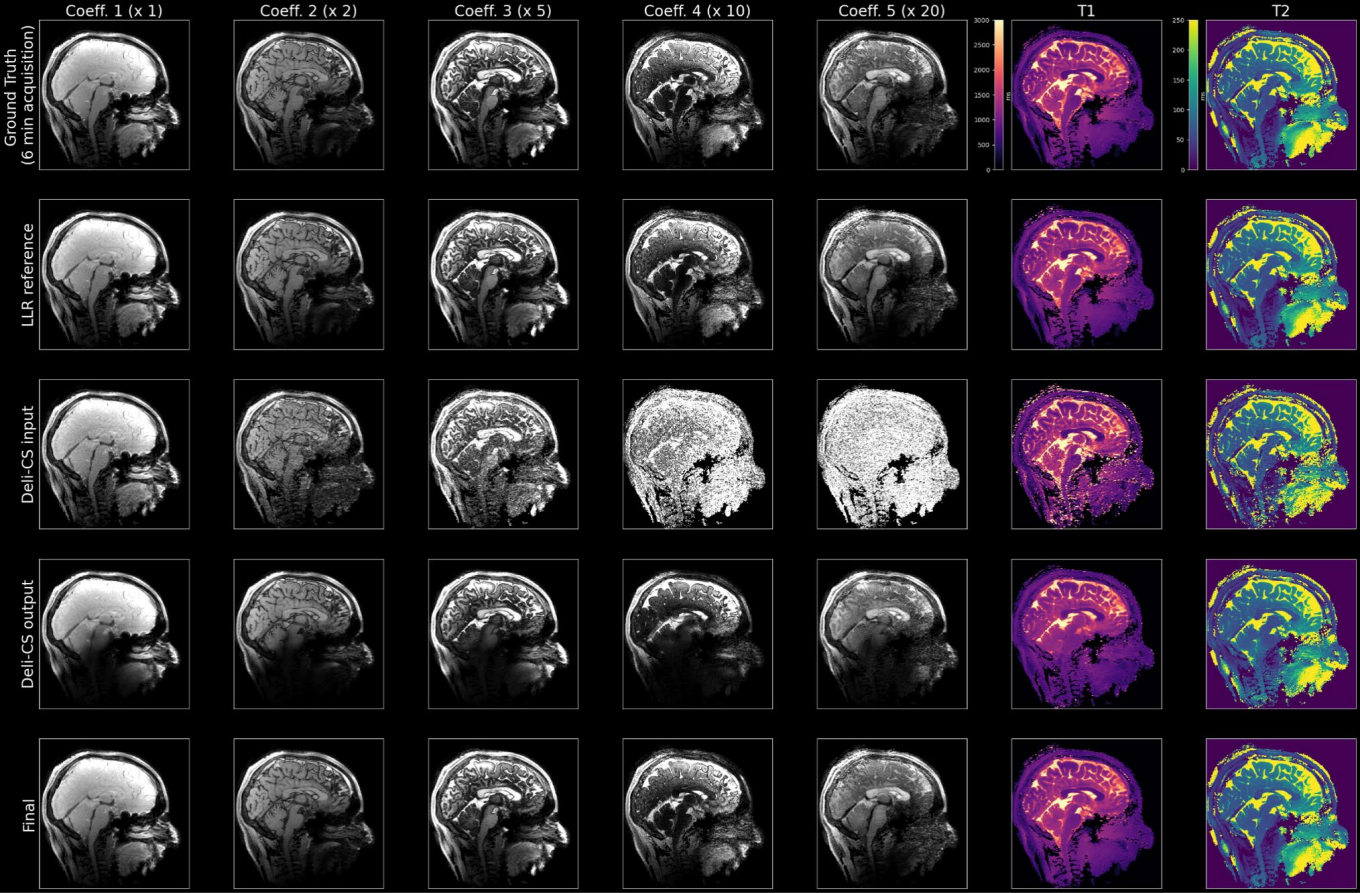
This figure compares the coefficient sagittal images recovered from the TGAS-SPI-MRF data using various methods for one of the healthy test subject. The first row denotes the reference LLR reconstruction of the 6-min data acquisition, and the second row denotes the LLR reconstruction of the retrospectively under-sampled 2-min acquisition. The remaining rows depict the various steps of the Deli-CS pipeline. The third row shows the initial adjoint reconstruction, the fourth row shows the model inference and the fifth row shows the reconstruction after iterative refinement. The number above each column shows how much the intensity of each coefficient image has been multiplied for visualization

**Fig. 6:**
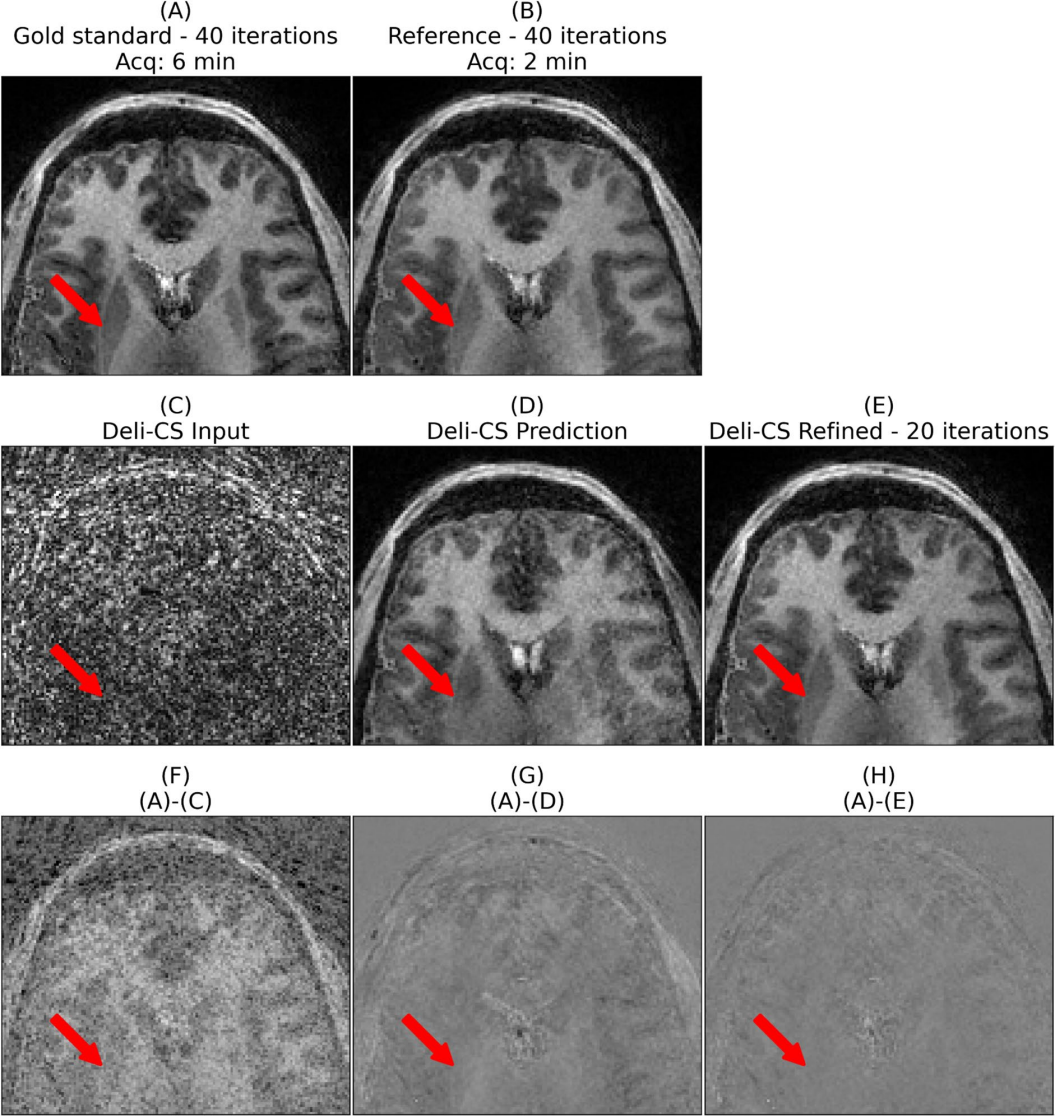
In this axial view of test subject 2 of the fifth (lowest energy) coefficient that contains much of the white and gray matter contrast of the putamen (denoted with a red arrow) is somewhat reduced using the Deli-CS inference but restored to the same level of contrast as the 2-min acquisition by the refinement step. It is also noticeable just how much denoising the network performs

**Fig. 7:**
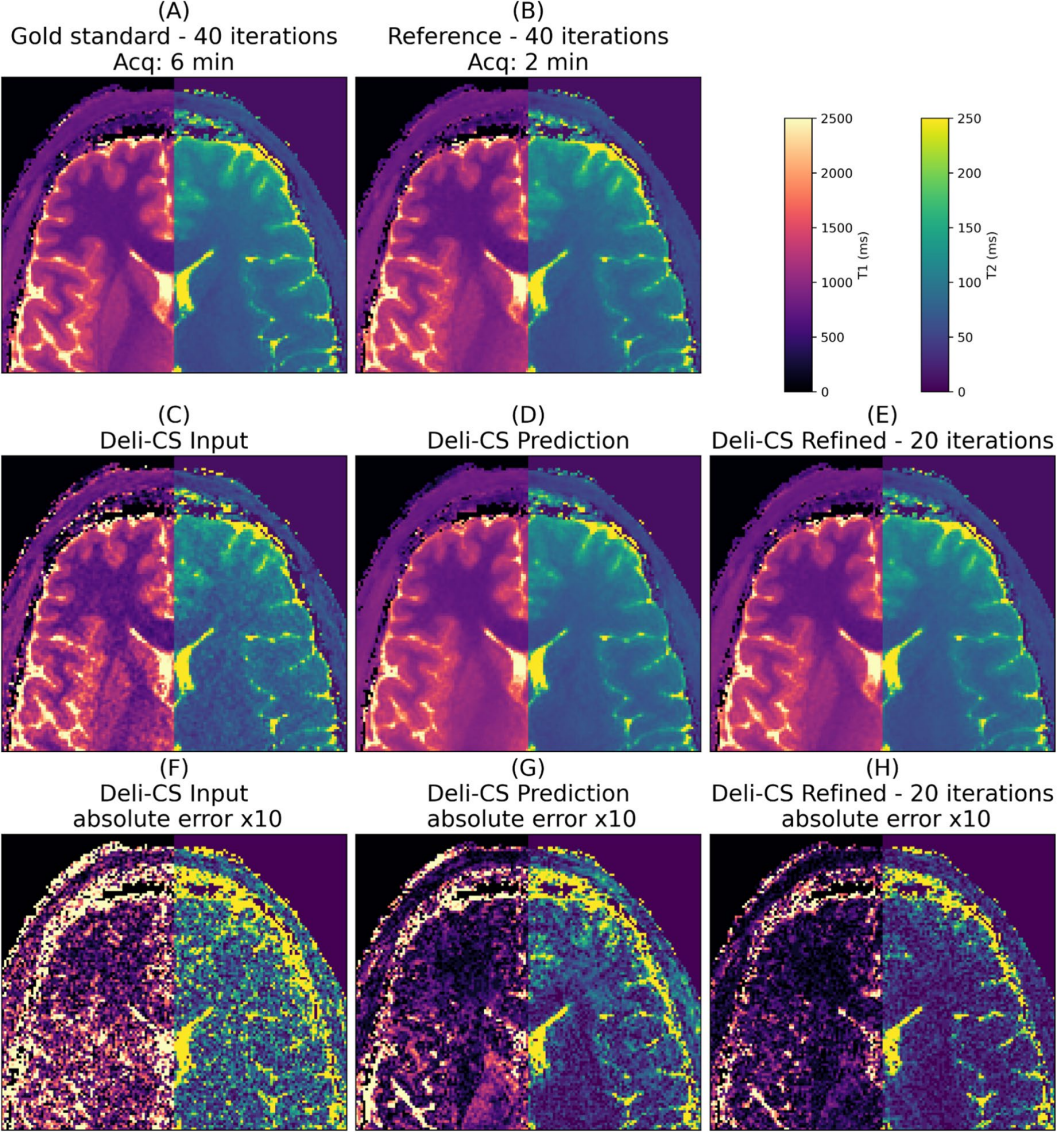
The quantitative maps of test subject 2 show high sharpness and contrast in both T1 and T2 maps with half the number of iterations when initialized with Deli-CS. T2 estimates benefit more than T1 estimates from Deli-CS initialization as they depend more on the lower energy coefficients

For a complete overview of the process, Fig. 5 shows a sagittal view of all steps within the reconstruction pipeline as well as the T1 and T2 quantification for one test subject. The DL step smoothed the image significantly, which can be seen more clearly in Figs. 6 and 7, and caused some bias in the quantitative values especially in low signal areas around the brain stem (Fig. 5). The refinement step retrieved more sharpness and correct quantitative values in the low signal areas. Comparing the Deli-CS input with its output shows improved quality in the lower energy fourth and fifth coefficients in particular.

As evidenced by Figs. 6 and 7 which show a more detailed view of the three steps of the process (Deli-CS input, output, and post refinement), the refinement step adds missing features that were over smoothed or misassigned from the network prediction. The detailed view of Fig. 6, shows just how much denoising the network performs on the lowest energy basis, but also that it can smooth out vital contrast information, such as the putamen, which the refinement step then recovers well. Dictionary fitting of the data, as shown in Fig. 7, on the other hand, shows that the fits from the Deli-CS input (the simple gridding reconstruction) result in noisy estimates, especially for the T2 map. Both the T2 and T1 map are strongly denoised by the Deli-CS network, and any contrast lost, such as the outline of the putamen as shown in the T1 map in the figure, is recovered by the refinement step.

**Fig. 8:**
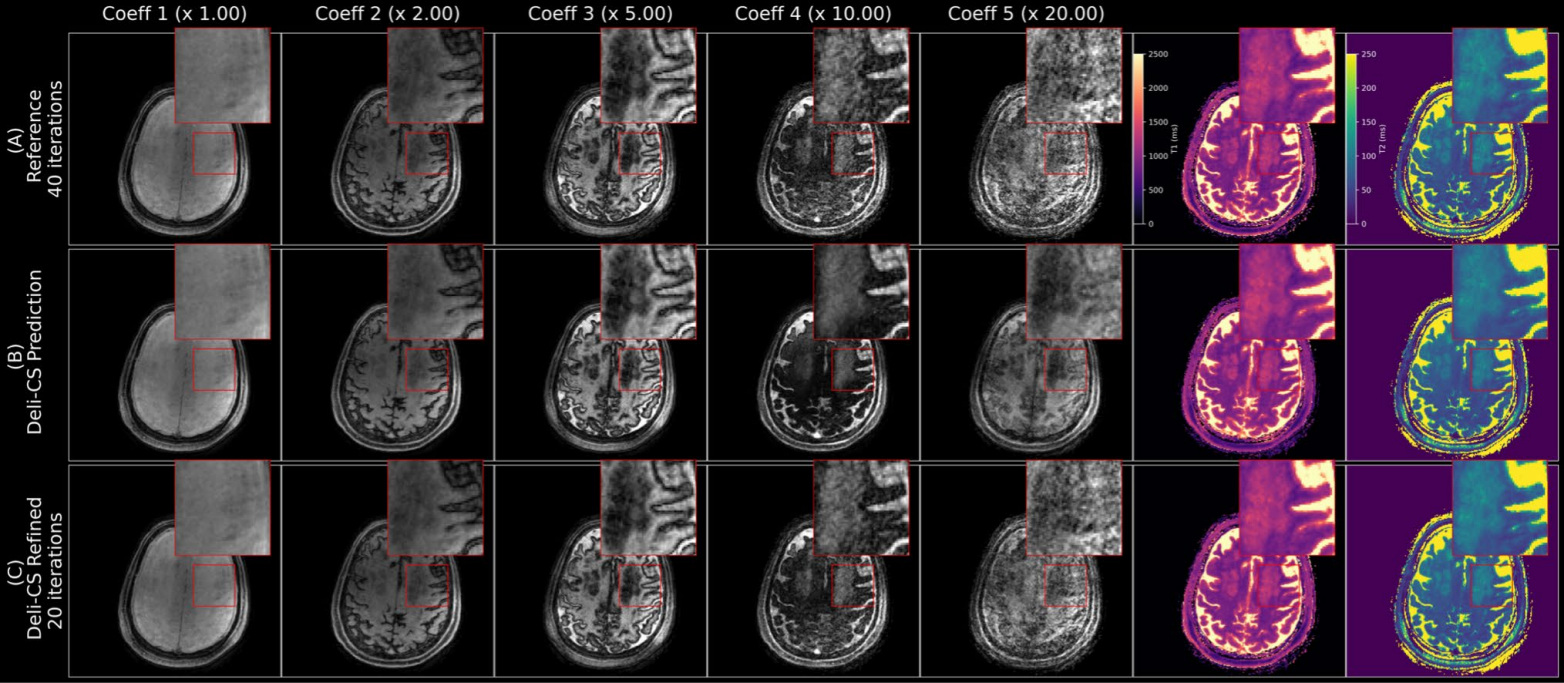
In this axial view of the subspace coefficients of a 75-year-old male patient with chronic small vessel disease that affects both T1 and T2 in the deep white matter of the bilateral centrum semiovale (left side shown in the inset) is clear. Even though the original data in the lower energy coefficients is very noisy, a clean estimate is generated by the Deli-CS prediction step and the refinement step removes any bias generated by the Deli-CS prediction and confirms consistency with the acquired data. The number above each column shows how much the intensity of each coefficient image has been multiplied for visualization. A more detailed view available in Fig. 9

**Fig. 9:**
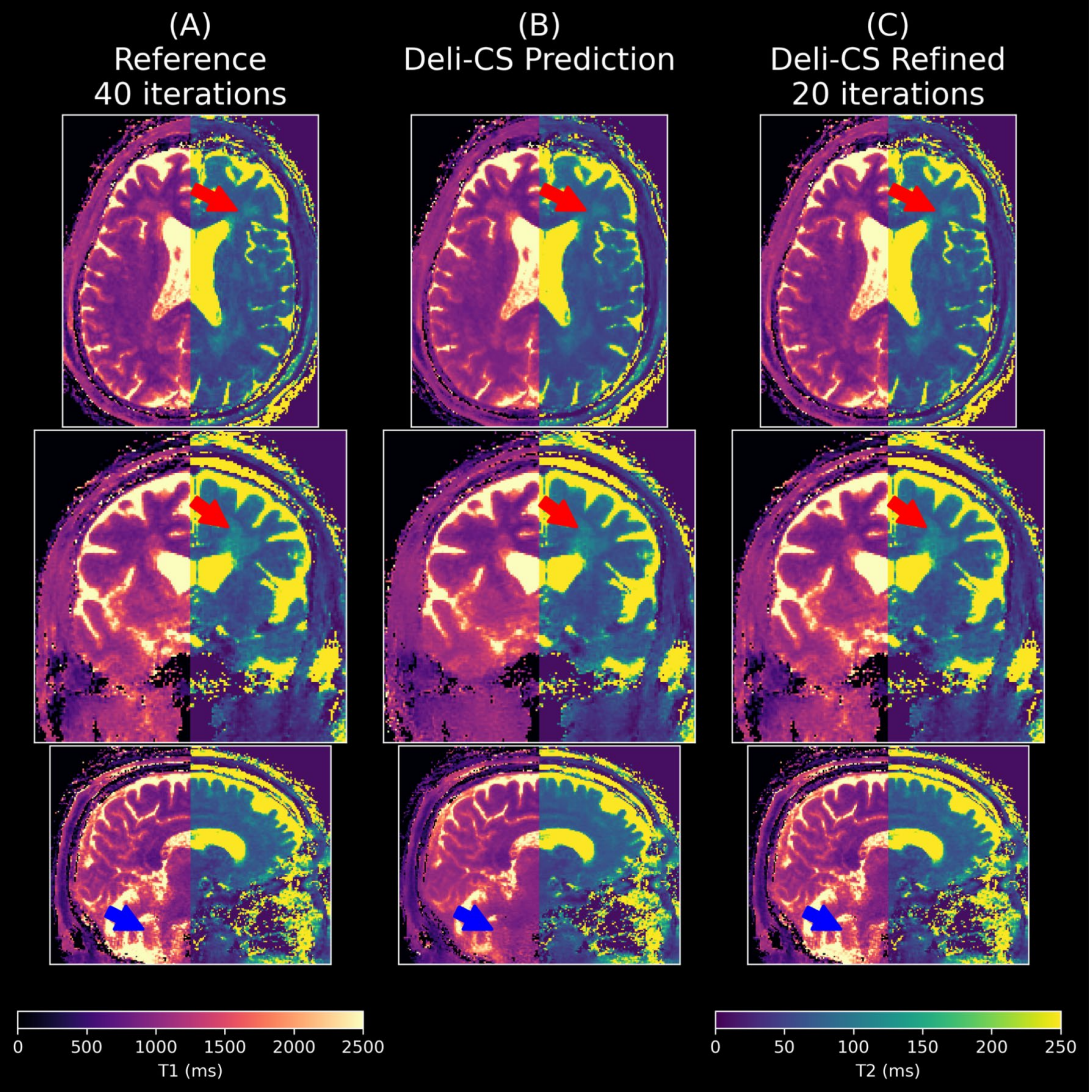
The refined quantitative maps in this patient (75 y/o male with chronic small vessel disease) show high sharpness and contrast in both T1 and T2 maps. Some areas are biased and blurred after the network inference (cerebellum: blue arrows, white matter lesion: red arrows), but recovered by the iterative refinement

**Fig. 10:**
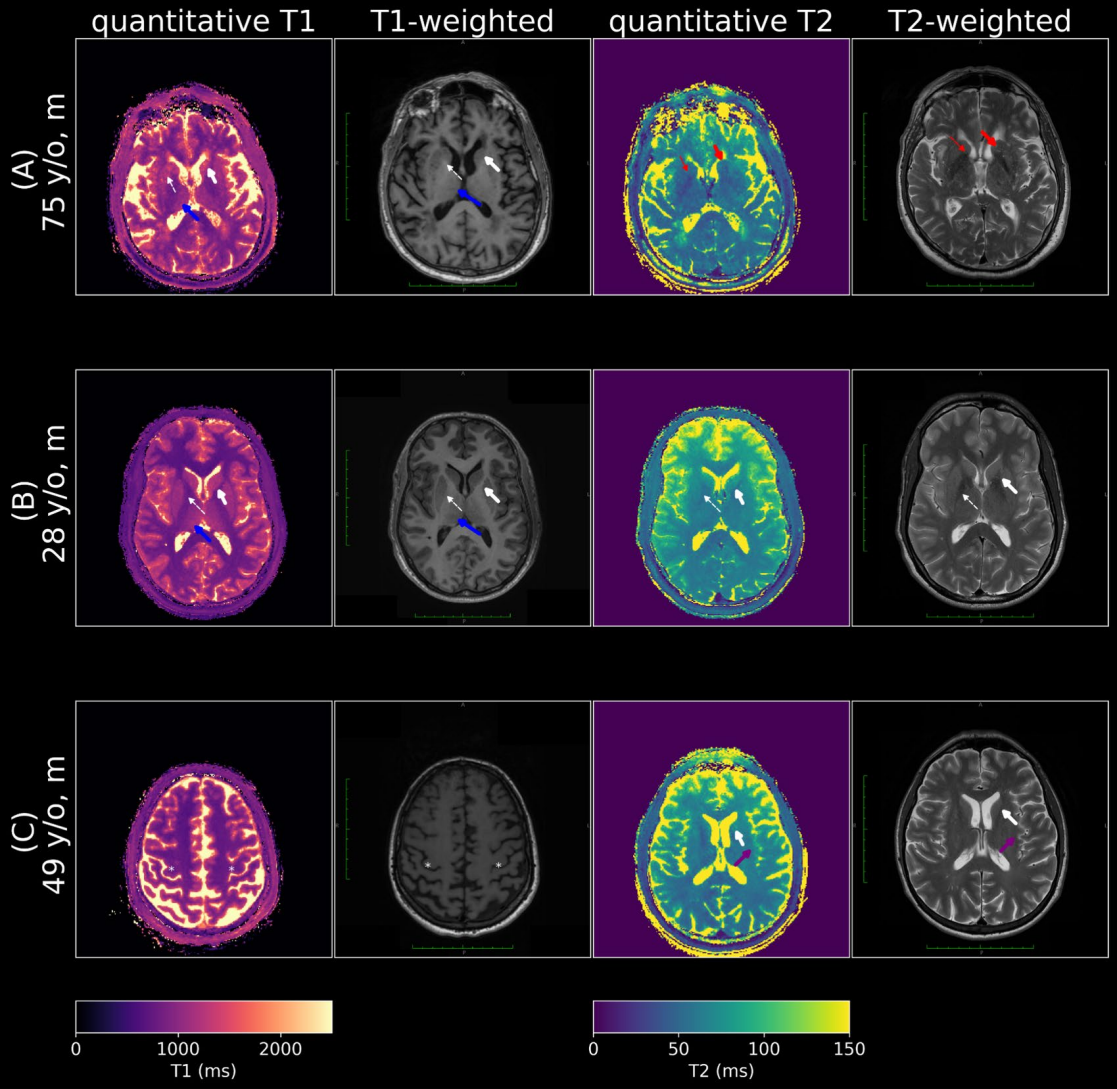
Comparison with standard clinical scans (slices approximately aligned). Note that T1 weighted images have inverted contrast to T1 maps as long T1 leads to low signal intensity on a T1 weighted image. In the T1 maps and T1 weighted images of **A** and **B** the caudate head (white arrow) and putamen (white dashed arrow) can be seen on the quantitative T1 map, but the delineation is slightly obscured compared to the T1 weighted image. The bilateral thalami (blue arrows) are more conspicuously delineated on the quantitative T1 map. The quantitative T1 map in (**C**) also demonstrates good gray-white distinction in the cerebrum as well as delineation of the hand-knob region (white asterisks). In (A) The striatum (red arrow) and globus pallidus (red dashed arrow) is somewhat obscured on the quantitative T2 map compared to the T2 weighted image. In **B**, the quantitative T2 map distinguishes the caudate head and putamen well and the borders of the ventricles are well-defined. In **C** the quantitative T2 map demonstrates clear borders of the ventricles. However, the definition of the caudate head and insula (purple arrow) are somewhat obscured

Similarly, in the patient data Figs. 8, 9, and 10), great improvements in denoising by the network and sharpness and contrast from the refinement step were observed. The baseline quality of the 2-min reference reconstruction was, however, lower than that of healthy volunteers. This is likely due to higher levels of motion as well as positioning further out of the head coil for patient comfort.

The 75-year-old patient had indications for chronic small vessel disease in both the T1 and T2 maps shown in Figs. 8, and 9, whereas the other two patients exhibited no significant clinical findings, consistent with their standard-of-care imaging protocol as shown in Fig. 10.

Despite not having trained on any data containing pathology, the Deli-CS network retained many clinically relevant features as shown in Fig. 10. Some deep gray matter areas that were visible on T2 weighted images were, however, obscured on quantitative T2 maps. This could be because the T2 weighted images have additional contrast from T1 and/or MT effects that are not captured in pure T2 maps [48].

## Discussion

This study introduces Deli-CS for MRI reconstruction, specifically for high-dimensional applications. Deli-CS uses a block-based deep learning approach to initialize the reconstruction process. In practical applications, this enables immediate assessments of scan quality using the fast intermediate steps of Deli-CS, allowing rescans if necessary. The refined reconstruction, taking an additional 20 min, can later be sent for detailed radiological assessment.

The new, GPU-optimized and sampling density compensated reconstruction method outperforms the prior reconstruction approach by Cao et al. [15] as evidenced by Fig. 3. However, we note that there are some residual minor streaking artifact left in the reconstruction both for the gold standard and the 2 min reconstructions (Fig. 6). In particular, some streaking is observed in the gold standard case. This is likely because we are looking at the 5th coefficient with 20 times smaller signal intensity than the first coefficient and small artifacts from imperfect acquisition (e.g. trajectory, motion etc.) could show up more clearly in this last coefficient.

The Deli-CS inference step denoises and smooths the initial reconstruction, and the refinement step suppresses hallucinations, bias in the parameter maps, and over-smoothing. In principle, the optimization in 5 has a unique solution that the iterations will converge to, regardless of initialization. We noticed that when initializing with A*b the iterations mainly denoise the result, whereas when initializing with Deli-CS they reduce biases in the quantitative parameter maps. The T2 maps benefit most from Deli-CS initialization as the T2 estimation is more dependent on the lower energy coefficient maps which suffer the most from noise and aliasing in the A*b initialization.

By being GPU efficient, the proposed framework is expected to scale well to ultra-high resolution sub-millimeter applications, or higher numbers of coefficients that could improve the accuracy of the quantification and encode more tissue parameters, e.g. diffusion parameters such as in [49]. Additionally, enforcing a minimal amount of GPU memory for training simplifies the process of *continuous training* to update the learned model to account for potential distribution shifts, which is an important factor to consider.

when deploying deep learning methods [50–54].

In the patient data, the denoising effect of the DL prediction step was very strong and the Deli-CS predictions before the refinement looked much cleaner in terms of noise and tissue contrast than both the reference image and subsequent refinement step. But although the reconstruction was clean there is no way to certify whether or not the DL method hallucinated features that were not actually there and whether the clean-looking image was consistent with the data acquired as no data consistency is included in this step. As unrolled methods get more powerful and multi-GPU solutions become more readily available, the Deli-CS prediction can be used as a prior instead of a starting point in iterative reconstructions and thus be useful in improving image quality as well as reconstruction times.

The good recovery of the fourth and fifth coefficients in the healthy volunteer data is also expected to improve more advanced quantitative parameter fitting methods. In particular, since a voxel typically consists of multiple tissue types, the better-resolved fourth and fifth coefficients are expected to allow for the better fitting of multiple T1 and T2 values per voxel.

While this work aims to describe a general deep learning initialization approach, prior similar work [34, 55] have proposed DL for rapid whole brain reconstruction. The proposed Deli-CS frameworks aims to fully leverage MRI physics and differs from Gomez et al. [55] by fully leveraging subspace and coil information to enable robust reconstruction at high accelerations which leads to shorter scan times. The deep learning prediction is used to initialize an iterative reconstruction instead of being used as the output of the framework similar to Wang et al. [34], which guards against potential hallucinations and improves robustness as shown above in Figs. 6 and 7. Although the idea of using DL as a warm start to a compressed sensing reconstruction, is the same as proposed by Wang et al., the novelty of Deli-CS lies in applying such an approach to large 3D MRF reconstruction with subspace reconstruction and its implementation.

The six-minute TGAS-SPI-MRF reconstruction was used as a reference for assessing the method, but not for training. There were two reasons for this: 1. Without an independent ground truth it is challenging to compare the two-minute pipelines (no DL initialization versus DeliCS) to each other. And 2, by training on the 2-min data, allows for expanding the training data set more easily as the acquisition is short and can be added to clinical protocols such that patient data can be used for training as well in expansions of this work.

Note that in this work no B1 or B0 correction was included. B1 + correction would enable more robust parametric mapping, and B0 information can be incorporated into the **A** matrix in Eq. 2 to alleviate blurring issues related to spiral imaging, particularly in regions where the B0-inhomogeneity is large [15]. This extension is a good fit for the Deli-CS framework, as the network can potentially learn to predict a B0 corrected image using the non-B0 corrected input or multiple frequency shifted inputs, which.

could result in fewer refinement iterations (that would use the full forward model **A** with incorporated B0). This is beneficial as **A** augmented with B0 is even more computationally challenging as discussed by Cao et al. [15], making the traditional LLR reconstruction even harder to perform using realistic time and hardware constraints.

Finally, for further speedup, it would be ideal to port Deli-CS to a more efficient compiled language, which is expected to provide at least another 2–3 × in speed improvement. Further acceleration could also be achieved by parallel processing on multiple GPUs [56], but for this project, a single GPU implementation was chosen for improved accessibility of the method. Further ablation studies can also be performed to optimize the network architecture and hyperparameters, for example implementing a complex-valued network [57] instead of splitting into real and imaginary components, to reduce the number of refinement iterations needed for convergence after Deli-CS inference.

## Conclusion

Deli-CS reduces the reconstruction time of subspace reconstruction of volumetric spatio-temporal acquisitions by providing a warm start to the iterative reconstruction algorithm. The DL-generated image, which has a risk of some bias and blurring, can be used for real-time quality assessment whilst the patient is in the scanner, whereas the refined reconstruction provides high-quality data in 20 min with minimal hardware requirements compared to the initial implementation of TGAS-SPI-MRF.

## Supplementary Information

Below is the link to the electronic supplementary material.

Supplementary file1 (DOCX 60 KB)

## Data Availability

The code and data used to generate the above results can be found in these repositories and public records: https://github.com/SetsompopLab/deli-cs, https://github.com/SetsompopLab/MRF, https://zenodo.org/record/7734431, https://zenodo.org/record/7703200, https://zenodo.org/record/7697373. The first repository link provides instructions on how to download and use the data and also links to the second repository, which contains the reconstruction code.
