# Supplementary material for: Deep learning initialized compressed sensing (Deli-CS) in volumetric spatio-temporal subspace reconstruction
Source: MAGMA. 2025 Feb 1;38(2):221–37. doi: 10.1007/s10334-024-01222-2 (PMC11914339; doi:10.1007/s10334-024-01222-2)
Supplement: Supplementary file 1 — Supplementary file1 (DOCX 60 KB) [file 10334_2024_1222_MOESM1_ESM.docx]

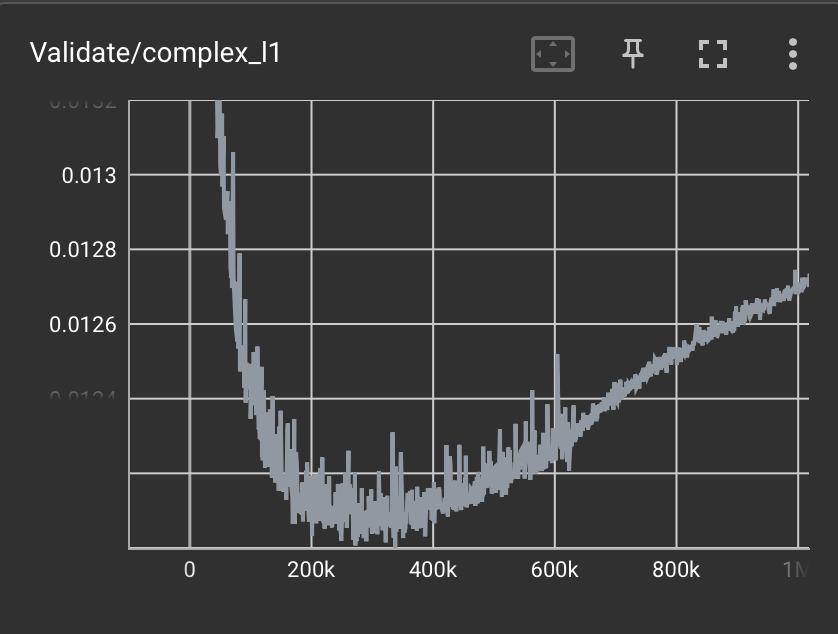


Figure S1 - L1 loss calculated on the validation set. The minimum is reached at step 337584, in epoch 540, after that the loss increases.
